# Bacterial diversity and geomicrobiology of Winter Wonderland ice cave, Utah, USA

**DOI:** 10.1002/mbo3.1426

**Published:** 2024-07-12

**Authors:** Miranda Herschel Seixas, Jeffrey S. Munroe, Erin M. Eggleston

**Affiliations:** ^1^ Department of Earth and Climate Sciences Middlebury College Middlebury Vermont USA; ^2^ Biology Department Middlebury College Middlebury Vermont USA

**Keywords:** cryogenic cave carbonates, geomicrobiology, ice cave, microbial ecology

## Abstract

The Winter Wonderland ice cave, located at an elevation of 3140 m above sea level in the Uinta Mountains of northern Utah, USA, maintains a constant sub‐zero temperature. Seasonal snowmelt and rain enter the cave, freeze on the surface of the existing ice, and contribute to a 3‐m‐thick layered ice mass. This ice mass contains organic matter and cryogenic cave carbonates (CCCs) that date back centuries. In this study, samples of ice, liquid water, and exposed CCCs were collected to examine the bacterial communities within the cave and to determine if these communities vary spatially and between sample types. Flow cytometry showed that cell counts are an order of magnitude higher in liquid water samples than in ice. Epifluorescence microscopy and scanning electron microscopy imaging revealed potential coccoid and bacillus microbial morphologies in water samples and putative cells or calcite spherules in the CCCs. The diversity of bacteria associated with soil, identified through sequence‐based analysis, supports the hypothesis that water enters the cave by filtering through soil and bedrock. A differential abundance of bacterial taxa was observed between sample types, with the greatest diversity found in CCCs. This supports a geomicrobiological framework where microbes aggregate in the water, sink into a concentrated layer, and precipitate out of the ice with the CCCs, thereby reducing the cell counts in the ice. These CCCs may provide essential nutrients for the bacteria or could themselves be products of biomineralization.

## Graphical abstract

The initial findings on bacterial diversity and geochemical data indicate that microbes enter the Winter Wonderland ice cave from the epikarst, carried by seasonal meltwater. The presence of these microbes in water, cryogenic cave carbonates (CCCs), and ice suggests they originate from the soil and are preserved differently depending on the sample type within the cave system. Notable differences exist between the water and CCCs, as well as observable potential cells or calcite spherules. These findings warrant further investigation into the possibility of biological mineralization in this system.

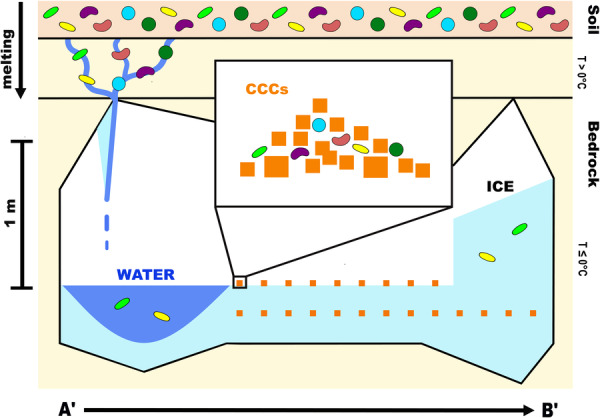

## 1 INTRODUCTION

Earth's cryosphere is anywhere that water is at least seasonally present as ice, including terrestrial ice sheets, mountain glaciers, polar marine ice shelves, and sea ice (Boetius et al., 2015). The cryosphere makes up about one‐fifth of the planet's surface, however, these environments are in rapid decline due to the effects of global climate change (Boetius et al., 2015; Shukla et al., 2019). Within the cryosphere are ice caves, which are environments with mean annual air temperatures below 0°C. This is often attributed to the geometry of cave entrances, which allow cold and dense winter air to sink into the cave where it remains during the summer months (Brad et al., 2018; Higham & Palmer, 2018; Luetscher & Jeannin, 2004; O'Connor et al., 2021; Perșoiu & Onac, 2019).

Ice caves are unique and understudied environments typically found in karst and alpine settings, but also in lava tube systems (Brad et al., 2018; Higham & Palmer, 2018; Luetscher & Jeannin, 2004; O'Connor et al., 2021; Perșoiu & Onac, 2019). Ice caves located just below the permafrost table can receive seasonal inputs of liquid water through the epikarst, the region above the cave that includes soil and bedrock. This water then freezes after reaching the subzero cave interior (Bakalowicz, 2019). Ice caves often lack precipitated speleothems (i.e., calcite or gypsum), but instead have other characteristic features, such as stratified ice blocks and cryogenic cave carbonates (CCCs), which provide records of terrestrial processes, atmospheric conditions, and regional temperature fluctuations (Brad et al., 2018; Faimon et al., 2012; Higham & Palmer, 2018; Holmlund et al., 2005; Kern & Perşoiu, 2013; Luetscher & Jeannin, 2004; Munroe, 2021; Perșoiu & Onac, 2019; Žák et al., 2008, 2012).

CCCs precipitate when water containing sufficient dissolved solutes enters the cave and freezes in the subzero environment (Žák et al., 2008, 2012). Previous work (Žák et al., 2008) identified two types of CCCs, CCC_coarse_ and CCC_fine_, distinguished based on their stable isotope values; CCC_coarse_ has lower values of δ^18^O compared with CCC_fine_ (Luetscher et al., 2013; Žák et al., 2012). CCC_coarse_ precipitates in closed system pools, which form when permafrost degradation allows drip water to enter a cave and collect on the ice surface, which then slowly freeze. CCC_coarse_ is considered a specific paleoclimate proxy marking episodes of permafrost thaw (Wong & Breecker, 2015; Žák et al., 2012). In contrast, CCC_fine_ precipitates from a thin layer of water on top of existing ice and has ambiguous climatic significance. CCCs have been shown to precipitate in distinctly spherical structures based on water chemistry (Tracy et al., 1998). Although the geochemical controls of CCC formation have been studied extensively, the potential role of CCCs as a nutrient source for ice cave microbial communities or inversely, the role of microorganisms in the biomineralization of CCCs, have not been investigated.

Cold‐adapted microbes throughout the cryosphere, and cave microbes around the world, have been studied to understand complex metabolic and geochemical processes, yet ice caves specifically are understudied microbiologically (Barton, 2006; Engel et al., 2010; Fenice, 2016; Hoover & Pikuta, 2010; Jones, 2001; Murray et al., 2012; Priscu et al., 1998; Purcarea, 2018). This is in part due to the inaccessibility of most ice caves, which are often found in geographically remote areas and at high elevations, requiring long hikes and technical approaches to access, which places constraints on sampling. The low latitude of the continental United States and the dry climate in many of its mountain regions contribute to a particular scarcity of known ice caves. As of 2018, there were only 15 known major ice caves in the United States, the majority of which are in the western states (Higham & Palmer, 2018). The most well‐studied ice caves in North America are lava tube ice caves (Higham & Palmer, 2018; O'Connor et al., 2021; Popa et al., 2012; Teehera et al., 2018). Ice caves in Canada have been more thoroughly studied structurally and chemically than those at lower latitudes, but the full extent of published research on microbes in ice caves in North America is limited (Black, 1954; Yonge & Macdonald, 1999). In contrast, European ice caves have been studied in much greater depth (Holmlund et al., 2005). The first identification of ice cave microorganisms was in 1949 in the Scărișoara Cave, Romania (Purcarea, 2018). The first ice cave bacterial strain, sampled from the Austrian alpine ice cave Eisriesenwelt, was isolated in 2003 (Margesin et al., 2004). 16S and 18S ribosomal RNA (rRNA) techniques were used in an ice cave in the Pyrenees to characterize the microbial community in an ice core dating back 5000 years ago (Ruiz‐Blas et al., 2023). Microbial communities in the Scărișoara ice cave were further investigated for spatial and temporal differences corresponding with differing sample ages, light exposures, and organic contents. Results indicate that the source of the water from which the ice froze, much more than the chemistry of the ice, determined bacterial functional diversity (Paun et al., 2019). Additionally, as the age of the ice increased, the abundance and viability of the cells decreased (Itcus et al., 2018). These results suggest that the source water for the ice and the age of the ice influence the microbial community, more so than the water chemistry.

Microorganisms have a diverse range of physical and metabolic adaptations that enable them to survive in extreme temperature conditions (Boetius et al., 2015; Margesin et al., 2007). At the same time, through metabolic processes, microorganisms can microscopically adapt their environment (Andrews, 2017). An example of this is biomineralization, in which living organisms produce minerals, either by direct synthesis within cells or outside the microbe as a result of metabolic products (Dhami et al., 2013). Microbially induced calcite precipitation is a biomineralization process that occurs in environments rich in Ca^2+^ ions, such as limestone caves (Dhami et al., 2013; Enyedi et al., 2020; Liu et al., 2018; Paul et al., 2017; Rusznyák et al., 2012). The ability to precipitate calcite is common among both soil bacteria and cave microorganisms (Barton & Northup, 2007). Moreover, microbially‐induced calcite precipitation, often dominated by the phyla Actinobacteria, Firmicutes, and Proteobacteria, has been shown to occur in geographically disparate limestone caves in Hungary, Germany, and China (Lange‐Enyedi et al., 2022; Liu et al., 2018; Rusznyák et al., 2012). The first microbial investigation of CCCs in Hawaiian lava tube ice caves found a diverse community dominated by Actinobacteria and Proteobacteria, microorganisms with the genetic potential for calcite precipitation (Teehera et al., 2018). Microbial communities in CCCs are vastly understudied; additional work is needed to better understand the role biomineralization might play in CCC precipitation.

Herein, we build upon prior physical and chemical investigations of Winter Wonderland Ice Cave (WWIC) to provide the first taxonomic characterization of microorganisms from an alpine ice cave in North America and the first microbial description of CCCs from a limestone cave in North America. This is a critical time to understand ice cave environments as many are undergoing significant melting due to the effects of global climate change (Cavicchioli et al., 2019; Fuhrmann, 2007; Kern & Perşoiu, 2013; Kern & Thomas, 2014; Pflitsch et al., 2016; Veni et al., 2014). This research adds to the understanding and feasibility of microbial paleoclimate records available in ice caves and creates a geomicrobiological framework for WWIC—a baseline from which to monitor the effect of anthropogenic climate change on ice cave microorganisms in North America.

## 2 MATERIALS AND METHODS

### 2.1 Site description

**Figure 1:**
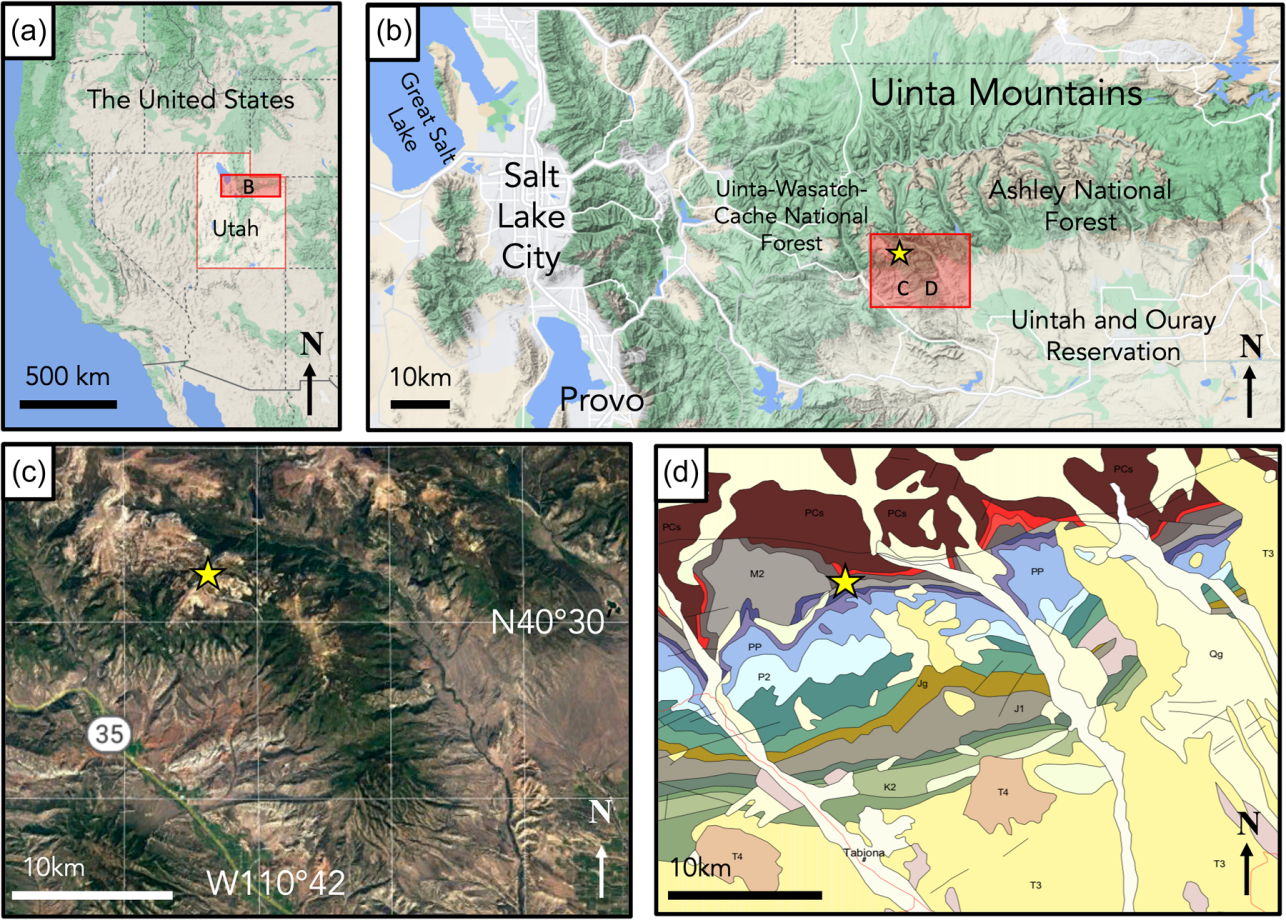
Location of the study area. The yellow star indicates the site of the Winter Wonderland Ice Cave. Maps of (a) Utah situated in the western United States, (b) the east‐west trending Uinta Mountain Range (c) the specific region of the Uinta Mountains where WWIC is located, and (d) the bedrock geology of Utah showing WWIC within Mississippian Madison Limestone (M1, dark gray) surrounded by Pennsylvanian, Mississippian, and Proterozoic limestones, shales, and sandstones (Hintze, 1980). The part labels a–c are adapted from Google Maps images accessed in 2020. WWIC, Winter Wonderland Ice Cave.

**Figure 2:**
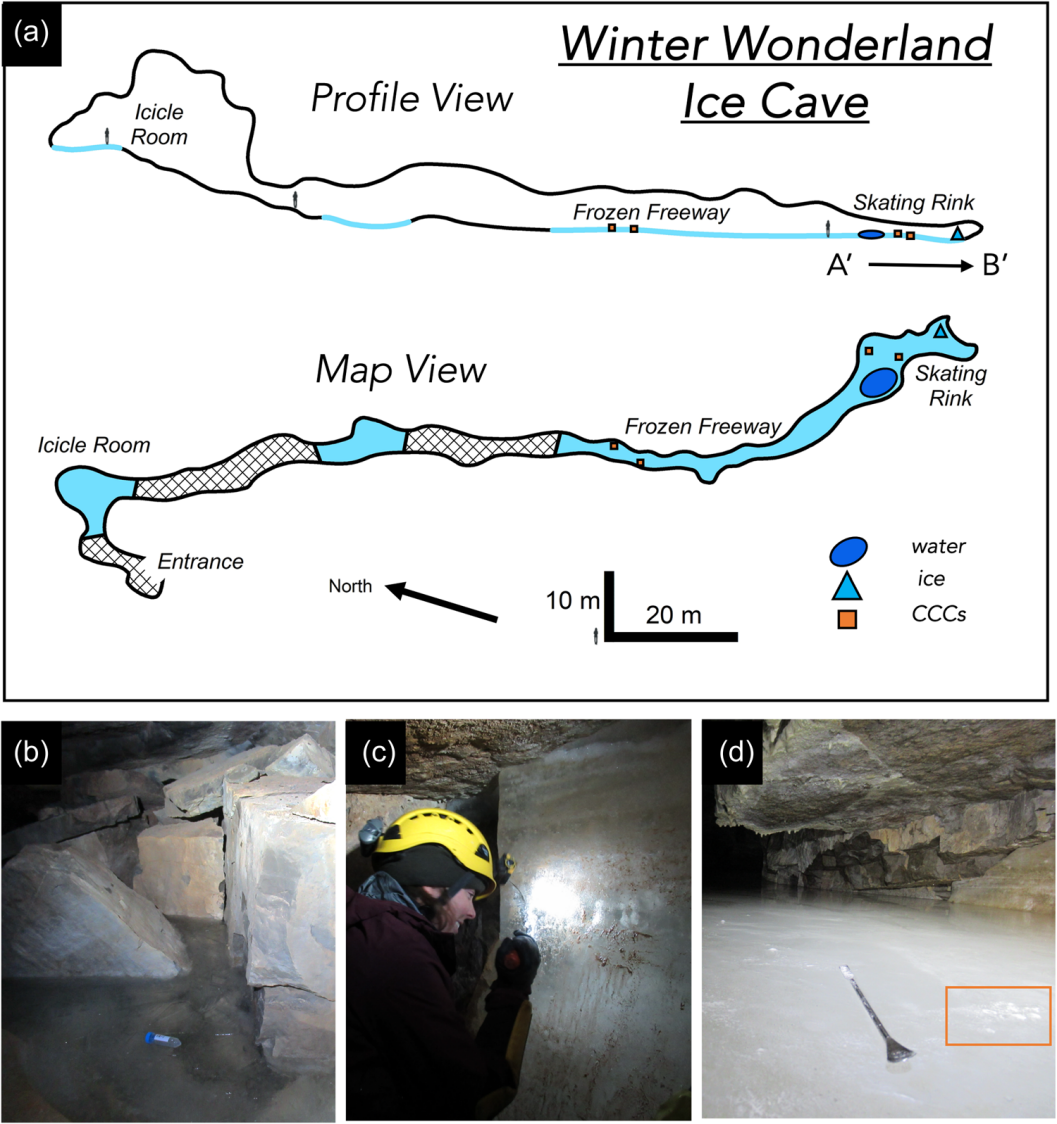
Profile and map view of the Winter Wonderland Ice Cave with sampling locations. (a) Profile and plan view of the cave. The A’ to B’ transect is used for orientation in Figure 6. (b) A shallow layer of water on the surface of the Skating Rink, with a 50 mL sample tube for scale, (c) an ice wall at the back of the Skating Rink, from which the ice block was removed, and (d) CCCs (white) within the orange box on the surface of the ice in the Skating Room. A 10‐cm long spatula for scale. Cave map adapted from Munroe (2021). CCC, cryogenic cave carbonate.

Identified by cave researchers with the U.S. Forest Service in 2014, Winter Wonderland ice cave (WWIC) in the Uinta Mountains, Utah, USA is one of the few limestone‐hosted ice caves described in North America (Figure 1). Located within Carboniferous‐Age Madison Limestone ~4 km south of Stillwater Reservoir above South Fork Rock Creek, the cave has a north‐facing entrance at an elevation of 3140 m above sea level, and a 245‐m long vadose passage, divided into three distinct rooms: the Icicle Room, Frozen Freeway, and Skating Rink with a maximum height of 33 m (Figure 2). Temperatures in WWIC maintain <0°C year round (Munroe, 2021). Air movement within the cave suggests the existence of a chimney‐like conduit connecting to the surface plateau 100 m above (Munroe, 2021). Either through this connection or through smaller fractures in the bedrock, solute‐rich meltwater enters the cave in the summer, freezing and precipitating CCCs, forming a laminar ice body that dates back multiple centuries and records CCC precipitation events (Munroe, 2021). WWIC is the first location in the western hemisphere where CCC_coarse_ has been reported, although CCC_fine_ are also present (Munroe, Kimble, et al., 2021). X‐ray diffraction (XRD) analysis has shown the CCC_coarse_ in WWIC to be predominantly composed of calcite with higher levels of magnesium, whereas the CCC_fine_ contain calcite, quartz, and detrital sedimentary material, suggesting that there may be small‐scale variability in available nutrients within the CCCs (Munroe, Kimble, et al., 2021).

### 2.2 Sample collection

WWIC was visited for this project on 19 August 2019. Ice, water, and CCC samples were collected while wearing nitrile gloves and from areas of the cave untouched by our field team. Samples were collected primarily from the Skating Rink, the region farthest from the cave entrance (Figure 2), which at this time was covered by a thin layer of liquid water. Three 50 mL water samples were collected in sterile tubes from a pool about 3 × 1 × 0.5 m with a thin lid of ice on the southwest edge of the Skating Rink. A 30 × 15 × 15 cm block of ice was cut from the ice wall at the back of the Skating Rink using a drywall saw. The ice block was transported intact and in the dark from Utah to Vermont in a liquid nitrogen dry shipper, then stored at −80°C until processing. CCC samples were collected using an alcohol‐sterilized spatula and chisel. For each sample, an approximately 4 × 4 cm layer of carbonate minerals on the surface of the ice was scraped into a 50 mL Falcon tube. A total of 11 CCC samples were collected in 2019 from the same locations as CCC samples from 2018 (Munroe, Kimble, et al., 2021). In the Skating Rink, 5 samples were collected from the south side of the room (“Skating Rink Right”) and 3 from the north side (“Skating Rink Left”). For spatial comparison, samples were collected from the Frozen Freeway as well; 2 CCC samples from the north side (“Frozen Freeway Left”) and 1 from the south side (“Frozen Freeway Right”) (Figure 2, Map View). Water and CCC samples were kept in the dark in a cooler with ice during transport. CCCs were placed in a −20°C freezer and water samples were placed in a 4°C refrigerator until they were filtered about 1 week after sample collection.

### 2.3 Sample cleaning

Cleaning of the ice block for microbial analyzes was performed inside a class 10 clean bench within a cold room at the Osterberg Lab in the Earth Sciences department at Dartmouth College, Hanover, NH. The bench used a Clean Rooms International HEPA filter (SAM MicroSound GS). Ceramic (ZrO) blades were left soaking in a 5% Citranox® solution for 3 weeks, then rinsed with MilliQ water. The stage and grips were also cleaned with the Citranox® solution. About 0.5 cm of ice was shaved using the ceramic blades, following ice‐core decontamination protocols (Schwikowski et al., 1999). The cleaned sample was then placed into acid‐washed autoclaved beakers and covered with parafilm. This cleaned ice sample, as well as the environmentally contaminated outer shavings, were transported back to Middlebury on dry ice. Following a modified ice core melting protocol from Osterberg et al., 2006 the ice sample was melted on a hot plate at 25°C before sample allotment for flow cytometry (FCM) and filtration for DNA extraction.

### 2.4 Stable isotope analysis

Water samples were collected for stable isotope analysis (δ^18^O and δD) in 50 mL Falcon tubes with no head space, then filtered through 25 mm 0.2‐µm MCE membrane filters into new 50 mL tubes. These were analyzed <48 h later at Brigham Young University using a Los Gatos Research DLT‐100 water isotope analyzer with a CTC Analytics autosampler. Each sample was run eight times, along with a set of three standards, which were calibrated against VSMOW (Vienna Standard Mean Ocean Water). The precision of the resulting δ^18^O and *δ*D measurements is ±0.2‰ and ±1.0‰, respectively. Results were compared with stable isotope measurements of water and ice samples collected from WWIC in 2018 (Munroe, Kimble, et al., 2021) and monthly isotope values for the WWIC location predicated by the Online Isotopes in Precipitation Calculator (OIPC) (Bowen et al., 2005; Bowen, 2022; Welker, 2000).

### 2.5 Crystallography

Crystallography was performed in a cold room at the Cold Regions Research and Engineering Laboratory by cutting thin sections (1–2 mm) of intact ice samples using an electric saw. A Rigsby station was used to measure and image grain orientation to better understand how the ice formed and how microbes may be selectively preserved in the ice (Holmlund et al., 2005; Langway, 1958). A horizontal cross‐section provided a top‐down view, and two vertical cross‐sections provided a side view of the ice structure. These were melted and thinned on a lab benchtop to about 0.2 cm, then refrozen onto the sample holder, in preparation for crystallographic analyzes. A1/A5 and A2 values were recorded for an assortment of randomly chosen crystal grains, providing c‐axis data. Poles to the c‐axis were plotted using Stereonet 10 (Allmendinger, 2020).

### 2.6 Scanning electron microscopy (SEM)

A Tescan Vega 3 LMU scanning electron microscope coupled with an Oxford X‐Max 50 energy dispersive x‐ray spectrometer (SEM‐EDS) was used to analyze CCCs chemically and visually. CCC samples were dried overnight in an oven at 105°C, mixed to homogenize, and then adhered with conductive carbon adhesive tape to aluminum stubs each with an area of 10 cm^2^. Samples used to obtain high‐resolution images of CCC morphologies were covered with a conductive layer of gold‐palladium using an Ernest Fullam Inc. EffaCoater Au‐Pd Sputter Coater. Samples for elemental analysis were coated with carbon using an EmiTech K950X carbon evaporator and analyzed using EDS. AztecOne software was used for capturing backscatter electron images and performing elemental analyzes. The presence/absence of elements was determined by SEM‐EDS detection/lack of detection during randomized sampling of homogenized CCC samples.

### 2.7 Epifluorescence microscopy

Water samples were analyzed using epifluorescence microscopy to obtain data on cell abundance and morphological diversity (Santibáñez et al., 2016). Water samples were fixed with 10% v/v buffered formalin and vacuum filtered through 0.2 µm Polycarbonate Track Etched Membrane black disc filters using a filter funnel and a filter manifold with a 0.8 µm backing filter. The filters were allowed to dry before and after staining with SYBR Green I DNA stain, mounted in an antifade solution composed of 50% glycerol, 50% phosphate saline buffered solution (120 mM NaCl, 10 mM NaH_2_PO_4_, pH 7.5), and 0.1% *p*‐phenylenediamine, and observed at ×100 total magnification (Noble & Fuhrman, 1998; Patel et al., 2007).

### 2.8 Flow cytometry

FCM was performed on the water and ice samples. FCM background controls included muddy water from the melting of ice in the cave due to body heat and uncleaned portions of ice broken off from the block during transport, cleaning, and crystallographic analysis. A Beckman Coulter CytoFlex flow cytometer at the University of Vermont was used to quantify the cells in the water and melted ice samples (Marie et al., 1997; Paun et al., 2019; Santibáñez et al., 2016). MilliQ water was used as a negative control. Samples were stained for 30 min using a 10^−4^ concentration of SYBR Green I DNA stain before running through the flow cytometer. Bacterial counts used the sum of RNA and DNA fluorescence for each event due to SYBR Green I strong affinity for double stranded DNA (dsDNA), single‐stranded DNA, and RNA (Marie et al., 1997). A flow rate of 60 µL/min was used. Background noise varied from 13 to 20 events/µL, which is higher than normal, therefore conservative gating was used. This likely resulted in a slight underestimation of bacterial events in favor of excluding noise. A Shapiro–Wilk test confirmed that the data are not normally distributed, so a subsequent Kruskal–Wallis test (“tidyverse” package, RStudio) and Post Hoc Dunn Test (“FSA” package, RStudio) were used to compare counts among sample matrices (Wickham et al., 2019).

### 2.9 Microbial DNA extraction and 16S rRNA amplicon sequencing and analysis

Water, ice, and carbonate samples were analyzed using molecular microbial techniques (Mondini et al., 2018). Melted ice (600 mL) and water samples (50 mL) were filtered and concentrated using a 25 mm 0.22‐µm Millipore filter composed of mixed cellulose esters (CAT No. GSTF 025 00). DNA was extracted from water and ice (whole filter), and CCC samples (0.25 g per sample) using the ZYMO Quick‐DNA Fecal/Soil Microbe Microprep Kit (ZYMO Research) according to the manufacturer's instructions. Samples with low DNA concentrations were further concentrated using the ZYMO Genomic DNA Clean & Concentrator®−10. DNA was quantified using Invitrogen's Quant‐iT^TM^ PicoGreen^TM^ dsDNA Assay Kit and measured with an Applied Sciences QuantStudio 3 qPCR machine. All assays were assessed against a DNA standard curve (*R* ^2^ ≥ 0.99). Extracted DNA was used downstream with concentrations ≥0.33 ng/µL. Experimental samples (CCC, water, and ice) had a DNA concentration range of 0.43 to 23.14 ng/µL, with a median of 0.79 ng/µL. Sample controls had minimum and maximum values of 0.33 and 3.12 ng/µL, respectively. Amplicon libraries were generated using the ZYMO Quick‐16S NGS Library Prep Kit, which employs V3‐V4 archaeal and bacterial primers (Klindworth et al., 2013) according to the manufacturer's instructions. UltraPure™ DNase/RNase‐Free Distilled Water from Invitrogen™ was run through the ZYMO Quick‐16S NGS Library Prep Kit as a negative control (Salter et al., 2014). ZymoBIOMICS® Microbial Community DNA Standard (50 ng) was used as a reference mock community (positive control) by running this provided standard through the same protocol as the field samples. Using genetic barcodes to distinguish samples, a 16S rRNA library was created with 30 ng of DNA from each sample. This was sequenced at ZYMO Research Corporation in Irvine, CA, by their ZymoBIOMICS Service: Targeted Metagenomic Sequencing. Illumina MiSeq. A total of 300 bp paired‐end sequencing was performed with a spike of 10% PhiX. The quality of reverse reads was not sufficient for overlap between forward and reverse reads after trimming so further analysis was conducted on forward reads only. The dada2 pipeline was used to infer unique amplicon sequence variants (ASVs) from the raw reads (Callahan et al., 2016). ASVs were assigned taxonomic identities via DECIPHER based on the SILVA SSU release 138 database. Further data analysis, community composition, diversity metrics, and data visualization were conducted in R within RStudio using phyloseq v 1.36.0, ggplot2 v. 3.3.5 and additional packages. Alpha diversity was assessed between sample matrices using the Breakaway Betta model (Willis et al., 2017). Beta diversity between sample matrices was determined using Bray‐Curtis dissimilarity (Oksanen et al., 2022). Permutation multivariate analyzes of variance (PERMANOVA) with adonis2 were utilized to determine statistical significance between sample matrix Bray‐Curtis dissimilarity with the sum of squares modeling and 999 permutations (Oksanen et al., 2022). Differential ASV abundance analysis was conducted in corncob v. 0.3.1 with a false discovery rate *p*‐value cutoff of 0.05 (Martin et al., 2020).

## 3 RESULTS AND DISCUSSION

### 3.1 Geochemical and geophysical analysis

#### 3.1.1 Stable isotopes

Liquid water collected from the surface of the Skating Rink had average stable isotope values of −18.2‰ for *δ* ^18^O and −136.4‰ for *δ*D (Table 1). These stable oxygen isotope measurements of water samples are similar to values of November and April precipitation (Table 1 and Table A1) for the Uinta Mountains, as predicted by the OIPC (Bowen et al., 2005; Bowen, 2022; Munroe, 2021). However, in this environment, precipitation and meltwater can only reach the cave during summer months when the epikarst thaws. Additionally, as soon as liquid water reaches the cave it begins to freeze. If these samples, collected in liquid form during August, represent a precipitation event from the preceding April, then the rate of water transmission through the epikarst is very slow. Alternatively, the liquid entering WWIC in August may be a mixture of isotopically negative meltwater from spring snow and less negative summer precipitation, resulting in an isotopic signature coincidentally similar to April/November precipitation. A linear mixing model predicted contributions of 88% winter snowmelt and 12% summer rain in the water samples from 2019 (Munroe, 2021). Over time this balance between snowmelt and summer precipitation may have shifted. Previous stable isotope results from WWIC (Munroe, 2021) show that deeper, older samples within the layered ice deposit are more depleted in *δ* ^18^O and *δ*D. The ice block collected in 2019 from the exposed section of ice can be compared to the ice samples collected in 2018 (layers 7 and 8 from Munroe, 2021), which span the same layers of ice. These layers had *δ* ^18^O values of −13.6‰ and −12.7‰, respectively, and *δ*D values of −103‰ and −94‰, respectively (Table 1), less depleted in comparison to the water samples. The layered formation of the ice deposit in WWIC implies that the process of ice formation in WWIC begins with inputs of liquid entering the cave during warmer months which then freezes in distinct yearly layers. This process has operated for at least 500 years (Munroe, 2021). A similar process was reported for the Canyon Creek Ice Cave in Canada, where the isotopic composition of ice was shown to be linked to yearly snowmelt flood events (Yonge & Macdonald, 1999).

**Table 1:** Stable isotope *δ* ^18^O and *δ*D measurements from water and ice samples.

Sample	Average *δ* ^18^O ‰	SD *δ* ^18^O ‰	Average *δ*D ‰	SD *δ*D ‰	Referenced study
WWIC water	−18.2	0.2	−136.4	1	Current study
WWIC layer 7	−13.6	0.6	−103	4	Munroe (2021)
WWIC ice layer 8	−12.7	1.3	−94	8	Munroe (2021)
OIPC average April precipitation	−17.4	–	−126	–	Bowen et al. (2005); Bowen (2022)

Abbreviations: OIPC, Online Isotopes in Precipitation Calculator; WWIC, Winter Wonderland Ice Cave.

#### 3.1.2 Crystallography

**Figure A1:**
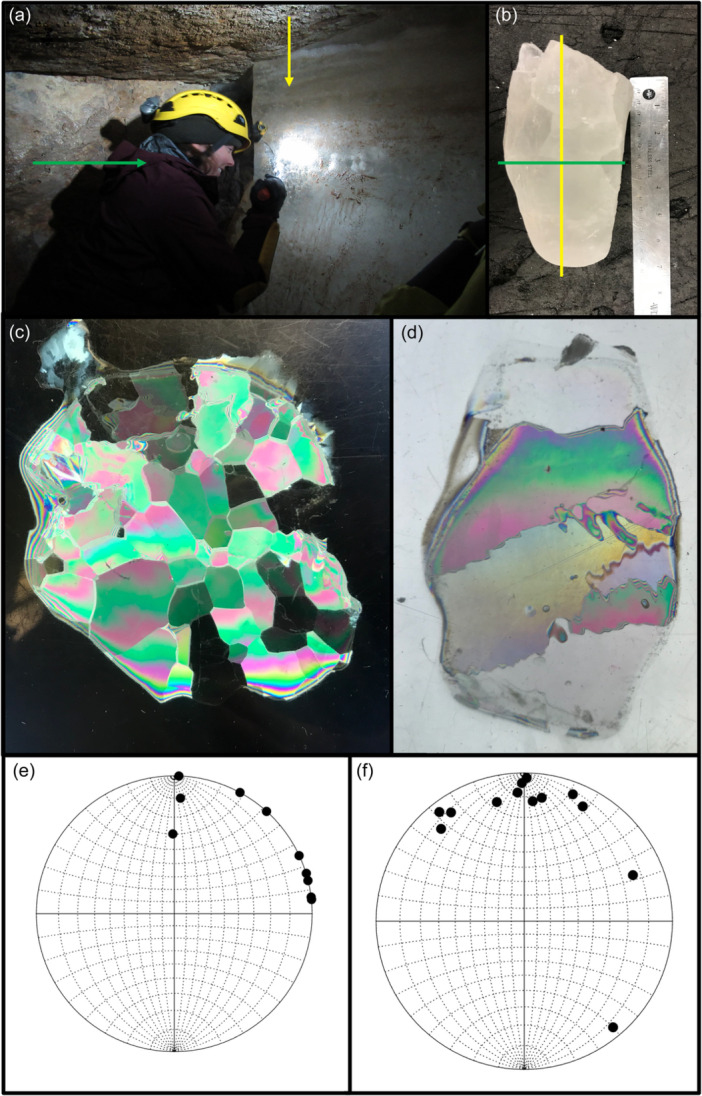
Crystallographic c‐axis data plotted on a stereonet (e, f), matched to an orientation on the ice wall (a), the orientation of ice block (b) and thin sections under cross‐polarized light (c, d). Yellow corresponds to top down view and green corresponds to the side view.

Due to our limited supply of ice reserved for DNA sequencing, grain orientation measurement sample size was not sufficiently large for statistical analysis, yet did provide qualitative results. Thin section analysis revealed vertically elongated crystal grains, with horizontal c‐axis mineral structures under cross‐polarized light (Figure A1). This pattern is commonly observed in freshwater environments when ice grows under calm conditions (Knight, 1962; Michel & Ramseier, 1971), particularly when the water is supercooled or seeded with preexisting ice crystals (Gow, 1986; Müller‐Stoffels et al., 2009). A top‐down view of the ice showed relatively equidimensional pseudo cubic hexagonal crystals between 2 and 6 mm in diameter (Figure A1). There is no indication that the ice in WWIC has been deformed by later movement or metamorphism, thus a lack of cells in the ice is more likely due to a process occurring before freezing, rather than the physiochemical rupturing of cells after freezing (Santibáñez et al., 2019; Žák et al., 2008).

#### 3.1.3 SEM elemental analysis

SEM‐EDS elemental analyzes from 2018 (Munroe et al., 2021) and 2019 (Table A1) confirmed that the CCCs identified in WWIC and in this study are predominantly composed of Ca‐carbonate. The most abundant elements are calcium, oxygen, and carbon, with varying amounts of silicon, aluminum, magnesium, potassium, phosphorus, iron, and sulfur (Table A1). The varying presence of these less dominant elements in the CCCs, perhaps sourced from detrital sedimentary material within CCC_fine_ (Munroe, Kimble, et al., 2021), may contribute to spatial differences in microbes across CCCs, providing much‐needed nutrients for microorganisms in this nutrient‐limited cave environment. Further work is needed to characterize this relationship.

#### 3.1.4 Scanning electron and epifluorescence microscopy

**Figure 3:**
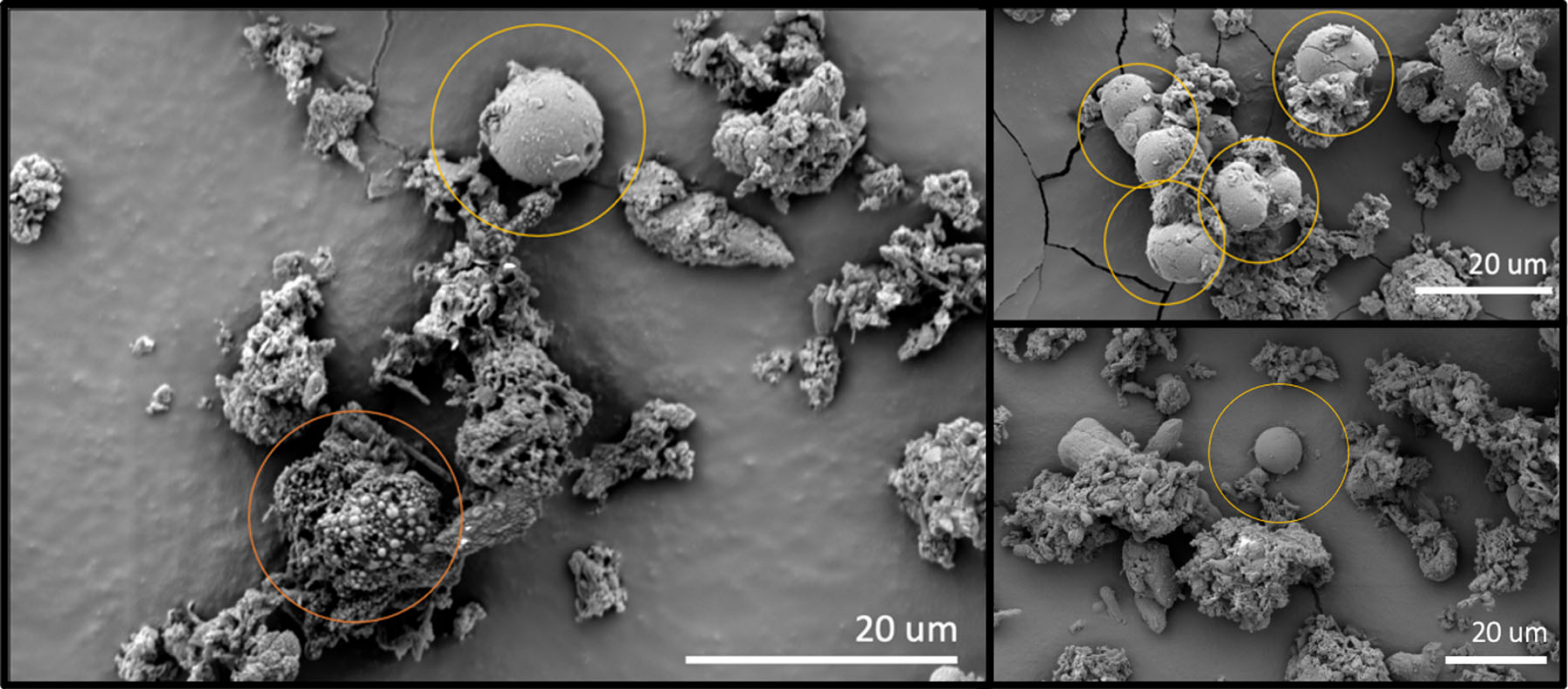
SEM images of CCCs coated with gold and palladium. Putative cellular aggregates or calcite spherules are denoted as 20 µm in diameter (yellow circles) and <2 µm in diameter (orange circles) within the crystal structure of the CCCs. CCC, cryogenic cave carbonate; SEM, scanning electron microscopy.

**Figure A2:**
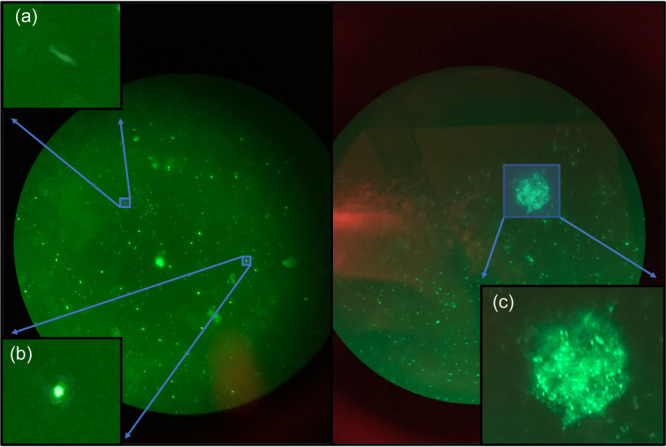
Fluorescence microscopy images of filtered water samples stained with SYBR Green 1. Note the insets of (a) a rod microorganism, (b) a cocci, and (c) an aggregate.

Epifluorescence microscopy of water samples revealed rods, cocci, and putative cellular aggregates in the water samples (Figure A2). SEM imagery was used to investigate microbes in CCCs—they were seen interspersed with uniform spherical shapes ~20 µm in diameter (Figure 3), which may be putative eukaryotic organisms, microbial aggregates, biomineralized calcareous structures, or calcite spherules (Figure 3 and Figure A2) (Cvetkovska et al., 2017; Dhami et al., 2013; Jantschke et al., 2020; Northup & Kathleen H. Lavoie, 2001; Paun et al., 2019; Tomczyk‐Żak & Zielenkiewicz, 2016). Eukaryotes are not detected with 16S rRNA sequencing, suggesting perhaps that this is not the explanation. Calcium was detected close to all these putative cells/calcite spherules in the CCCs, suggesting the biomineralization of calcareous structures (Table A1) (Dhami et al., 2013; Teehera et al., 2018; Tracy et al., 1998). Other studies have examined CCCs with SEM and have not identified putative cells (Lacelle et al., 2009; Spötl et al., 2023). However, these samples were not collected or stored using best practices for biological samples before visualization, perhaps resulting in the degradation of biological components.

### 3.2 Cell abundance and morphology—FCM

FCM revealed higher bacterial cell counts in water samples from the Skating Rink than the ice, background, and blank samples (Kruskal–Wallis rank sum test, *p*_adj._ = 0.004) (Table 2), with statistical significance driven by the difference between the background ice and the water samples (Post Hoc Dunn Test, *p*_adj._ = 0.03). Due to a pseudoreplicate of two, counts from the ice block could not be statistically analyzed, however cell counts were similar to those in the MilliQ blank. The ice backgrounds (unsterilized pieces of ice) had higher bacterial cell counts than the cleaned ice block, suggesting that there had been contamination of the outer layer of ice during transport. The lack of microorganisms in the clean ice as compared to the water samples may be due to a differential incorporation of bacteria and organic matter, which has been shown to occur during ice formation in lake ice (Santibáñez et al., 2019). Alternatively, the microbial cell membranes may rupture in the ice samples when the water enters the cave and freezes, the remnants being undetectable or discarded as noise during the FCM measurements (Muldrew & McGann, 1994). Even if SYBR Green I could stain these destroyed cells, they would resemble background noise and remain undetected by FCM. Another hypothesis is that the microorganisms percolating down from the epikarst may attach to sediments and particles in the water, forming aggregates, which were seen in fluorescence microscopy (Figure A2). These aggregates would be more likely to sink to the bottom of a pool of water and not be homogeneously mixed in as the water freezes (Simon et al., 2002). This would decrease the number of microbial cells that freeze into ice, except for specific layers where they would be more concentrated. It could be that the ice block that was sampled did not include any of these sediment‐rich and microbe‐rich layers. Alternatively, the microbial cells could attach to the carbonates as they begin to mineralize, precipitating out of the water together and decreasing the number of cells remaining in the water that freezes and becomes ice. This is supported by the SEM images of putative microbes attached to carbonate structures (Figure 3) and the diversity of organisms sequenced from CCCs.

**Table 2:** Flow cytometry measurements for average bacterial concentration in water and ice samples.

Sample	Average bacterial counts (events/µL)
Blank	21
**WWIC ice block**	**16**
Background ice	20
**WWIC water**	**396**
Background water	64

*Note*: Background samples are controls for possible contaminants introduced during sample collection, transport, cleaning, and processing.

Abbreviation: WWIC, Winter Wonderland Ice Cave.

### 3.3 Microbial community analysis

After removing chimeras, we obtained 5255 ASVs across all samples including CCCs (*n* = 10), cave ice (*n* = 1), and cave water (*n* = 3), as well as our controls including ice from processing the ice block sample (*n* = 2) and DNA extraction kit negative and positive controls. The median filtered, denoised, and nonchimeric reads per sample library was 16,618 reads, with the two outliers at the low end, one CCC sample library (1962 reads) and the kit negative control (7 reads). Only one ASV was removed due to its high frequency in the mock community positive DNA extraction control.

**Figure 4:**
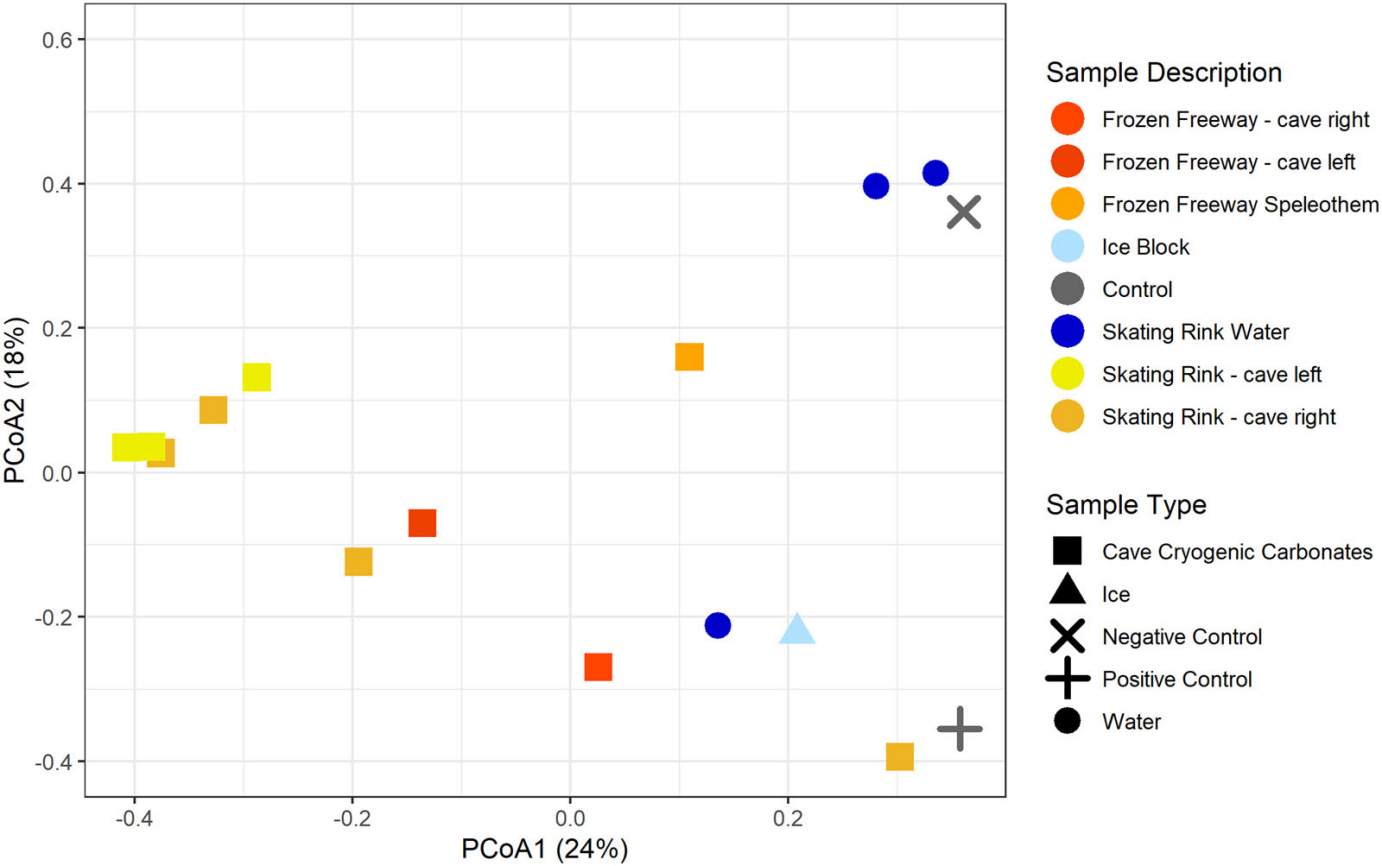
Principal coordinate analysis (PCoA) plots of the Bray‐Curtis dissimilarity of microbial communities from cave cryogenic carbonates, ice, water, and DNA extraction kit controls.

**Figure A3:**
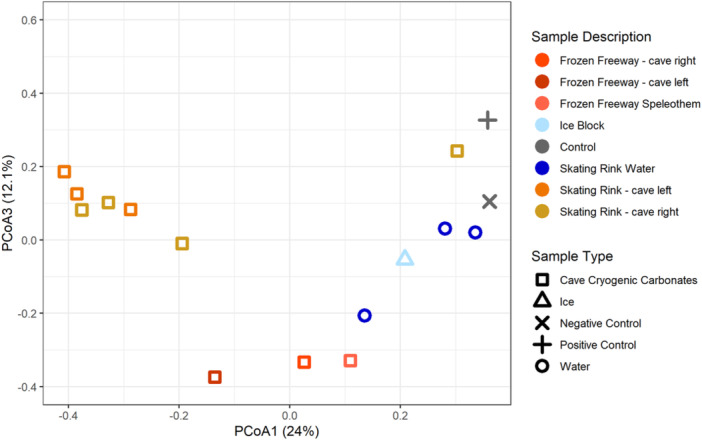
Principal coordinate analysis (PCoA) plots of the Bray‐Curtis dissimilarity of microbial communities from cave cryogenic carbonates, ice, water, and DNA extraction kit controls with the first and third most explanatory eigenvector axes.

Bray‐Curtis dissimilarity principal coordinate analysis reveals WWIC microbial community clustering (Figure 4). Principal coordinate analysis (PCoA) eigenvectors PCoA 1 and 2 were most explanatory for variance in these communities (24% and 18% variance, respectively) with the next eigenvalue (i.e., PCoA 3) explaining nearly equal variance (12.1%, Figure A3). Microbial communities associated with CCCs are tightly clustered with some separation in PCoA1 based upon cave location (i.e., Frozen Freeway and the Skating Rink) (Figure 4). Skating Rink CCCs cluster more distinctly than Frozen Freeway CCCs, perhaps due to the more isolated nature of the Skating Rink room. One water sample from the Skating Rink clusters with the ice block sample and two water samples cluster with the negative kit control (i.e., nuclease‐free water). The ice block microbial community appears unique from the CCC microbial communities, suggesting that as the water freezes, microorganisms are either incorporated into the ice or into the CCCs, and not preserved equally in both. The mock community positive control is distinct from the other libraries, except for the Skating Rink CCC sample which had low read coverage. PERMANOVA analysis with 999 permutations suggests that these sample type distinctions are not quite significant (Pseudo‐F: 1.8568; *p* = 0.011).

**Figure A4:**
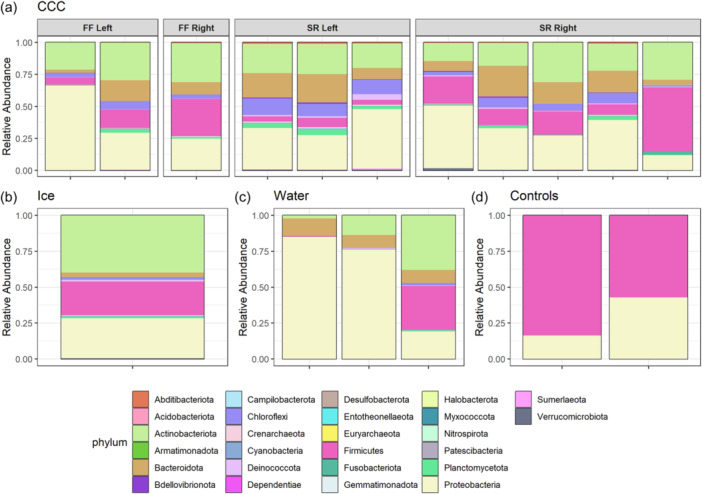
Stacked taxonomy plots by phylum for Frozen Freeway (FF) and Skating Rink (SR) CCCs (a), ice, (b), water (c) and positive and negative kit controls (d). Plots are scaled based on the abundance of ASV normalized by sample. CCC, cryogenic cave carbonate.

**Figure A5:**
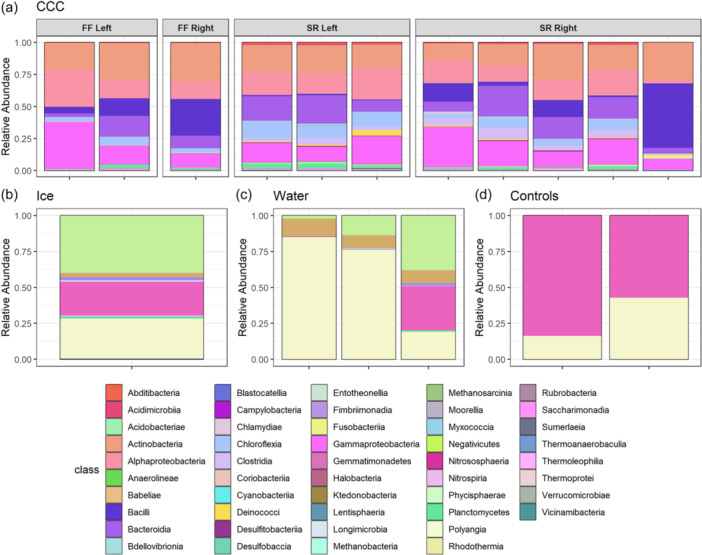
Stacked taxonomy plots by class for Frozen Freeway (FF) and Skating Rink (SR) CCCs (a), ice, (b), water (c) and positive and negative kit controls (d). Plots are scaled based on the abundance of ASV normalized by sample. CCC, cryogenic cave carbonate.

**Figure A6:**
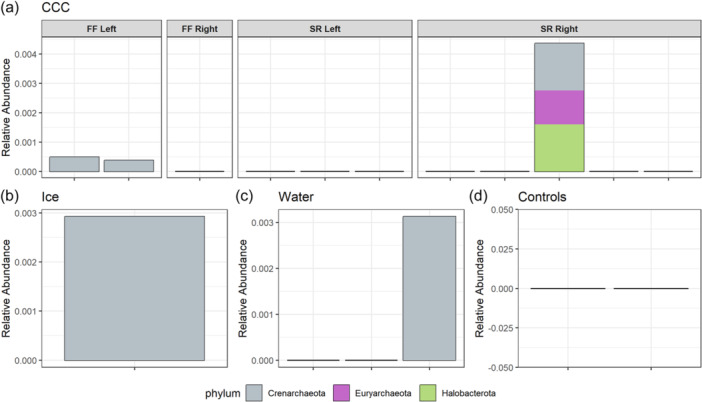
Subset of archaeal taxa for Frozen Freeway (FF) and Skating Rink (SR) CCCs (a), ice, (b), water (c) and positive and negative kit controls (d). Plots are scaled based on the abundance of ASV normalized by sample. CCC, cryogenic cave carbonate.

Microbial community compositional analyzes at the taxonomic levels of phylum and class show the diversity of taxa present in each sample type (Figures A4 and A5). Sequencing provided evidence of Actinobacteriota, Bacteriodota, Firmicutes, and Proteobacteria as the most dominant phyla (Figure A4) in all sample types with the most taxonomic richness of ASVs in the CCCs. Archaeal phyla were only present at very low relative abundance in some of the CCC samples, ice, and water (Figure A6). Actinobacteriota, Alphaproteobacteria, Bacilli, Bacteroidia, Chloroflexia, and Gammaproteobacteria were the dominant classes within CCCs (Figure A5). Estimates of species richness (i.e., ASVs) assessed with Breakaway betta (Willis et al., 2017) were highest in the CCC samples (323.5 ASVs ± 32.67), and showed a mix of phyla including Proteobacteria, Firmicutes, Chloroflexi, Bacteriodota, and Actinobacteriota (Figure A4). Intermediate species richness was estimated for our single ice sample (218.0 ASVs ± 122.24), with predominantly Proteobacteria, Firmicutes, and Actinobacteriota (Figure A4). Water samples had the lowest richness (109.7 ASVs ± 70.58) and had a range of Proteobacteria, Bacteriodota, and Actinobacteriota (Figure A4). Species richness ice estimates were not significantly different from the CCCs (*p* = 0.388).

Actinobacteria observed in all CCCs (Figure A5) are cosmopolitan and often dominant in cave environments (Buresova‐Faitova et al., 2022; Cuezva et al., 2012; Hathaway et al., 2014; Iţcuş et al., 2016; Lavoie et al., 2017; Riquelme et al., 2015; Teehera et al., 2018). They have been reported to be biocatalysts for mineral precipitation (Sanchez‐Moral et al., 2003). Cuezva et al., 2012 proposed that at low concentrations of CO_2_ or low humidity, Actinobacteria may precipitate crystallized CaCO_3_. Additional measurements from WWIC are needed to assess these environmental conditions. Alternatively, when organic matter in the environment is low Actinobacteria are thought to biogenically produce CaCO_3_ (Teehera et al., 2018). WWIC is very low in organic matter; that which is present is found concentrated at the cave entrance or deep within the ice (Munroe, 2021). Thus, the calcareous structures in the CCCs may be of biogenic origins produced by Actinobacteria.

**Figure 5:**
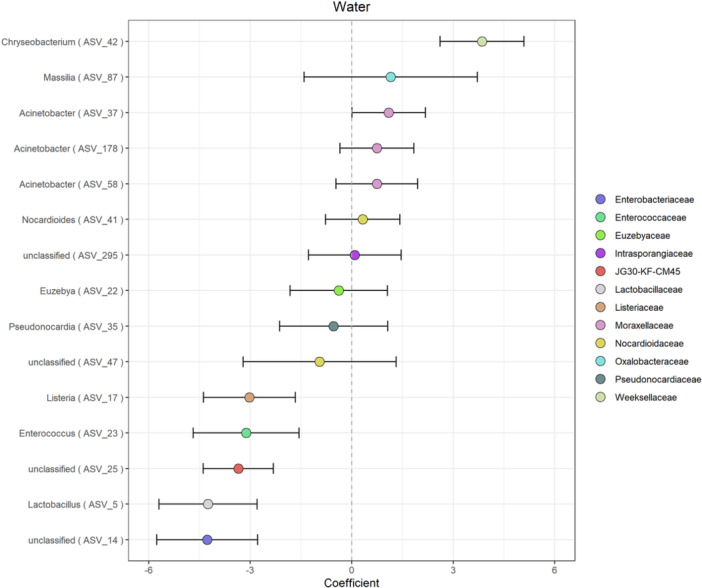
Differentially abundant amplicon sequence variants (ASVs) in water samples as compared to cryogenic cave carbonates. ASVs are plotted by genus and colored by family taxonomic levels. Taxa above zero are differentially enriched in water as compared to the CCCs. The false discovery rate cutoff is set at 0.05 to account for multiple comparisons (Martin et al., 2020). CCC, cryogenic cave carbonate.

Beta binomial regression analysis (Martin et al., 2020) revealed 13 differentially abundant ASVs in the water samples as compared to CCCs (PFDR = 0.05). CCCs were enriched in five ASVs as compared to water (*Lactobacillus*, *Listeria*, *Enterococcus*, and two unclassified genera) (Figure 5). While we only have one ice sample, these preliminary data suggest that CCCs were enriched in four ASVs as compared to ice (*Euzebya*, *Pseudonocarida*, and two unclassified genera). Water and ice samples were enriched in one ASV as compared to CCCs (*Chryseobacterium*). The ice sample was enriched in ASVs *Listeria*, *Enterococcus*, *Nocardiodes*, *Chryseobacterium*, *Massilia*, *Acinetobacter,* and one unclassified genus as compared to CCCs. Differential taxa identified are all bacterial not archaeal.

Putatively differentially abundant ASVs in the ice sample are consistent with previously described taxa from cold environments. *Massilia* spp. have been isolated previously from Antarctic waters and glacial streams (Gu et al., 2017; Holochová et al., 2020; Peeters et al., 2012; Sedláček et al., 2022; Shaffer et al., 2023). *Chryseobacterium* spp. have been isolated from ice cores in Greenland and Antarctica (Loveland‐Curtze et al., 2010; Raymond et al., 2008). Several *Listeria* species that are dominant in soil and water samples and are known to be cold‐adapted (Boetius et al., 2015; Linke et al., 2014) have been shown to adhere to abiotic surfaces in response to cold (Lee et al., 2017). Additionally, *Chryseobacterium* spp. have been isolated from ice cores in Greenland and Antarctica (Loveland‐Curtze et al., 2010; Raymond et al., 2008). This suggests that some of the microorganisms found within WWIC may be able to survive and adapt to the cold and dark conditions in the ice cave, especially if they are gaining nutrients from the CCCs. Further work will be necessary to further identify and test the metabolic activity of these microbes.

**Figure A7:**
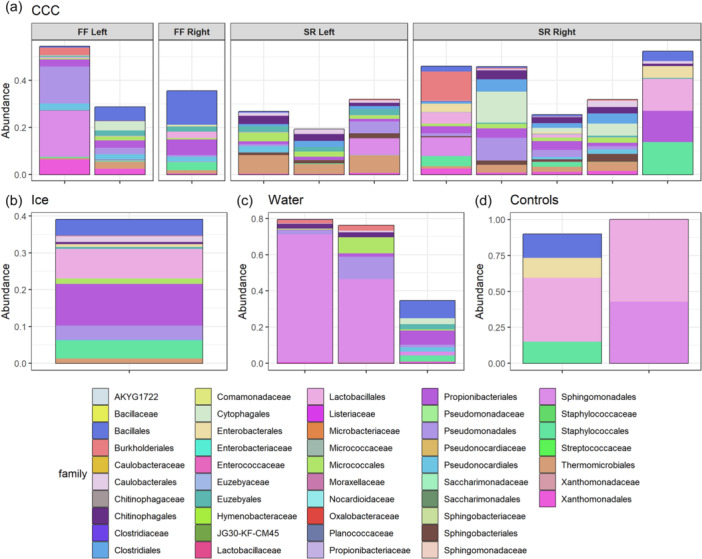
Subset of taxonomy by the family for Frozen Freeway (FF) and Skating Rink (SR) CCCs (a), ice, (b), water (c) and positive and negative kit controls (d). Plots are scaled based on the abundance of ASV normalized by sample. These plots only include taxa that were >10% abundant in at least one sample type. CCC, cryogenic cave carbonate.

Dominant microorganisms at the family level found in the WWIC across all sample types (Figure A7) are commonly associated with environmental soils including *Bacillales* and *Thermomicrobiales* (Delgado‐Baquerizo et al., 2018). This suggests that the source of microbes could be from the soil above, which is transported down in precipitation and meltwater through the epikarst and to the cave. Although no studies have been conducted on soil microbes in the Uinta Mountains to allow for comparison, this trend has also been reported in the limestone Herrenberg Cave in Germany (Rusznyák et al., 2012), where the dominant cave microorganisms are soil‐associated, and in lava tube ice caves in Hawaii, where the ice cave microbial communities are similar to microbial communities in volcanic soil deposits, suggesting some connection between the soil and the cave below (Rusznyák et al., 2012; Teehera et al., 2018). Bray Curtis dissimilarity beta diversity (Figure 4) showed distinctions, although not quite statistically significant, in microbial communities between water, ice, and CCCs, and between CCCs sampled from different parts of the cave. CCCs had the most microbial diversity of all sample types. Skating Rink CCCs are more clustered, which could be due to the distance from the cave entrance, creating a relatively undisturbed environment. Spatial differences in communities across the CCC samples may be explained by fracture patterns in the bedrock above, allowing water to enter the ice cave at specific locations after traveling distinctly different paths (Barton, 2006). The source of water for each room in WWIC could also be different, resulting in distinct microbial communities, and influencing which microbes are available to precipitate out of the water with the CCCs. Given the narrow cave entrance (<25 cm) we suspect that there would be limited airborne contribution of microbes to the communities we sampled (Figure 2) in comparison to those from soil and water from the bedrock above.

## 4 CONCLUSIONS

**Figure 6:**
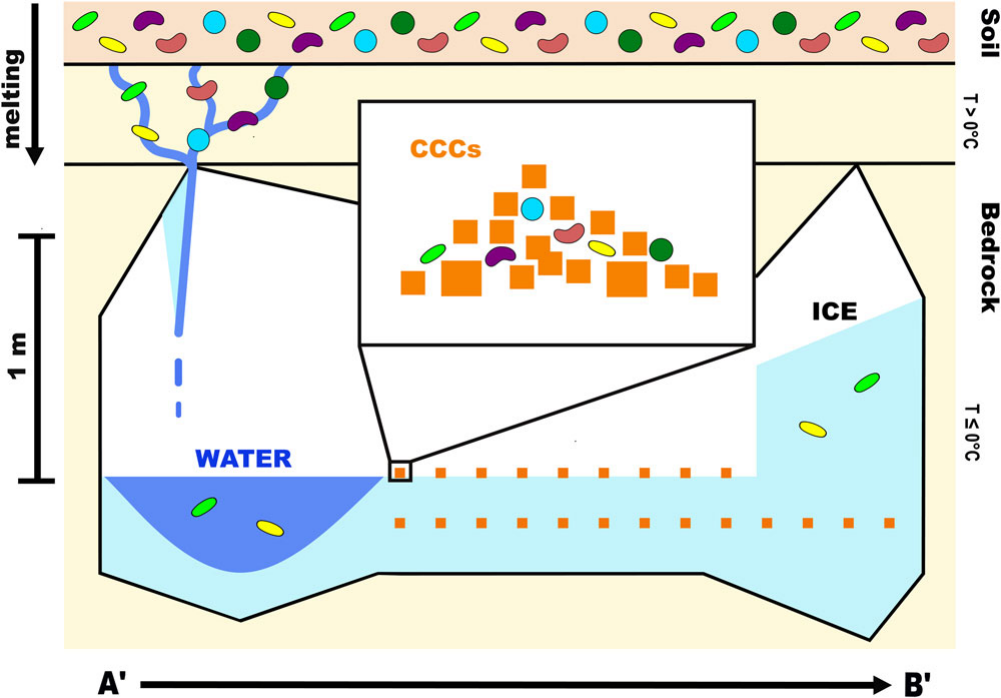
A geomicrobiological framework for Winter Wonderland Ice Cave, depicting microbial inputs and patterns of abundance and diversity in the Skating Rink of WWIC. Each colored shape represents an individual microorganism, with all 6 individuals representing a full soil microbiome. Sample types collected are water (dark blue), CCCs (orange squares), and ice (light blue). Soil is shown as light orange and limestone bedrock is yellow, with the permafrost table shown as a black line. A’ to B’ transect corresponds to the same transect in Figure 2. CCC, cryogenic cave carbonate; WWIC, Winter Wonderland Ice Cave.

Ice, water, and CCC samples from the WWIC in the Uinta Mountains, Utah were analyzed to better understand bacterial diversity in this unique environment and provide insights into the geomicrobiological processes in the cave. Stable isotope measurements suggest that the water entering the cave is a mixture of spring snow and summer rain (Table 1 and Table A1). Structural analysis of the ice determined that it has undergone little to no strain, suggesting that the absence of microbes in the ice is more likely due to preferential biotic incorporation, rather than physical rupturing of cells within the ice (Santibáñez et al., 2019). Based on our results, we propose a model (Figure 6) demonstrating the formation of water, ice, and CCCs and the preservation of microorganisms within those sample types. Microbial cells are most abundant in the liquid water samples from WWIC, suggesting that the water filtering through the epikarst and entering the cave during seasonal melt transports sediments, organic matter, and microbes. As the water pools on the surface of the ice and begins to freeze, microbial cells may aggregate or form biofilms as an adaptation to stress (Boetius et al., 2015). The heavier aggregates may collect at the bottom of the pool of water or attach to crystallizing CCCs. As the water fully freezes, the CCCs, associated cells, and aggregates concentrate into discrete visible layers rather than a homogenous mixture. These layers could be missed during randomized sampling. Additionally, some of the microorganisms entering the cave may be able to survive on nutrients contained within the CCCs, perhaps biomineralizing additional calcite structures. The preference of microorganisms for CCCs may result in the observed decrease in microbes within the ice (Figure 6). This differential incorporation of microbes during freezing likely creates variety in the number of microbes found between sample types. As a result, the cave ice alone may not accurately record the full microbial community present in the cave. This should be considered when using archival ice to understand microbial community changes and responses to environmental and climate change over long periods (McGrath, 2019; Singh et al., 2010; Wooliver et al., 2019).

## AUTHOR CONTRIBUTIONS

**Miranda Herschel Seixas**: conceptualization (equal); data curation (equal); formal analysis (equal); methodology (equal); writing—original draft (lead); writing—review & editing (equal). **Jeffrey S Munroe**: conceptualization (equal); formal analysis (equal); funding acquisition (lead); investigation (lead); methodology (equal); resources (lead); supervision (equal); writing—review & editing (supporting). **Erin M Eggleston**: conceptualization (equal); data curation (lead); formal analysis (equal); investigation (equal); methodology (equal); project administration (equal); supervision (equal); writing—original draft (supporting); writing—review & editing (lead).

## CONFLICT OF INTEREST STATEMENT

None declared.

## ETHICS STATEMENT

None required.

## Data Availability

Metadata and R code are available through GitHub (https://github.com/eme47/WWIC). Raw sequence data are available through the NCBI BioProject PRJNA1122174: https://www.ncbi.nlm.nih.gov/bioproject/PRJNA1122174.

## APPENDIX A

Table A2

**Table A1:** Presence/absence of elements in two CCC samples from WWIC as measured with a coupled SEM and EDS analysis.

C	O	Ca	Fe	Si	Mg	K	P	Al	S
+	+	+	+	+	+	+	+	+	−
+	+	+	−	+	+	−	+	+	+

Abbreviations: CCC, cryogenic cave carbonate; SEM, scanning electron microscopy; WWIC, Winter Wonderland Ice Cave.

**Table A2:** The Online Isotopes in Precipitation Calculator (OIPC), shows monthly averages for *δ*D and *δ* ^18^O at latitude 40.5° N, a longitude of 110.86° W, and an altitude of 3140 m.

	Jan	Feb	Mar	Apr	May	Jun	Jul	Aug	Sept	Oct	Nov	Dec
*δ*D	−173	−171	−153	−126	−102	−96	−62	−53	−73	−113	−143	−172
*δ* ^18^O	−23.1	−23.2	−21.2	−17.4	−14.5	−13.5	−9	−7.9	−10.4	−15.9	−19.4	−22.9
